# Poly[[hexa­aqua­tris­[μ_2_-2,5-dihy­droxy-1,4-benzoquinona­to(2−)]diholmium(III)] octa­deca­hydrate]

**DOI:** 10.1107/S1600536810028989

**Published:** 2010-09-25

**Authors:** Koji Nakabayashi, Shin-ichi Ohkoshi

**Affiliations:** a Department of Chemistry, School of Science, University of Tokyo, 7-3-1 Hongo, Bunkyo-Ku, Tokyo 113-0033, Japan

## Abstract

In the polymeric title compound, {[Ho_2_(C_6_H_2_O_4_)_3_(H_2_O)_6_]·18H_2_O}_*n*_, the Ho^III^ ion is nine-coordinated by six O atoms derived from three bidentate 2,5-dihy­droxy-1,4-benzoquinonate (DHBQ^2−^) ligands and three O atoms from three water mol­ecules. The Ho^III^ ions are connected *via* three ligands, resulting in the formation of a two-dimensional honeycomb layer parallel to the *ab* plane. The layer is racemic in which Δ- and Λ-coordination geometries around Ho^III^ ions are alternately arranged. The asymmetric unit comprises a third of a Ho^III^ ion, located on a threefold axis, one-half of a DHBQ^2−^ ion, located on a centre of inversion, one coordinated water mol­ecule and three uncoordinated water mol­ecules.

## Related literature

For general background, see: Kitagawa & Kawata (2002 ▶); Nakabayashi & Ohkoshi (2009 ▶); Ohkoshi *et al.* (2001 ▶). For details of the synthesis, see: Weider *et al.* (1985 ▶). For related structures, see: Robl & Sheldrick (1988 ▶); Weiss *et al.* (1986 ▶).
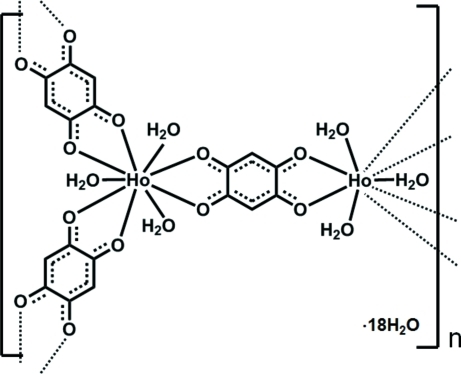


## Experimental

### 

#### Crystal data

[Ho_2_(C_6_H_2_O_4_)_3_(H_2_O)_6_]·18H_2_O
*M* *_r_* = 1176.46
Trigonal, 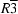

*a* = 14.1407 (3) Å
*c* = 18.0629 (5) Å
*V* = 3127.93 (12) Å^3^
*Z* = 3
Mo *K*α radiation
μ = 3.88 mm^−1^
*T* = 90 K
0.10 × 0.10 × 0.04 mm

#### Data collection

Rigaku R-AXIS RAPID diffractometer
Absorption correction: multi-scan (*ABSCOR*; Higashi, 1995 ▶) *T*_min_ = 0.704, *T*_max_ = 0.856
11262 measured reflections
1594 independent reflections
1525 reflections with *I* > 2σ(*I*)
*R*_int_ = 0.025

#### Refinement

*R*[*F* ^2^ > 2σ(*F* ^2^)] = 0.023
*wR*(*F* ^2^) = 0.063
*S* = 1.23
1594 reflections
86 parameters
H-atom parameters constrained
Δρ_max_ = 0.65 e Å^−3^
Δρ_min_ = −0.30 e Å^−3^

### 

Data collection: *PROCESS-AUTO* (Rigaku, 1998 ▶); cell refinement: *PROCESS-AUTO*; data reduction: *CrystalStructure* (Rigaku, 2007 ▶); program(s) used to solve structure: *SHELXS97* (Sheldrick, 2008 ▶); program(s) used to refine structure: *SHELXL97* (Sheldrick, 2008 ▶); molecular graphics: *ORTEP-3* (Farrugia, 1997 ▶) and *pyMOL* (DeLano, 2007 ▶); software used to prepare material for publication: *CrystalStructure*.

## Supplementary Material

Crystal structure: contains datablocks global, I. DOI: 10.1107/S1600536810028989/tk2691sup1.cif

Structure factors: contains datablocks I. DOI: 10.1107/S1600536810028989/tk2691Isup2.hkl

Additional supplementary materials:  crystallographic information; 3D view; checkCIF report

## Figures and Tables

**Table 1:** Selected bond lengths (Å)

Ho1—O1	2.371 (2)
Ho1—O2	2.463 (2)
Ho1—O3	2.385 (3)

**Fig. 1.**
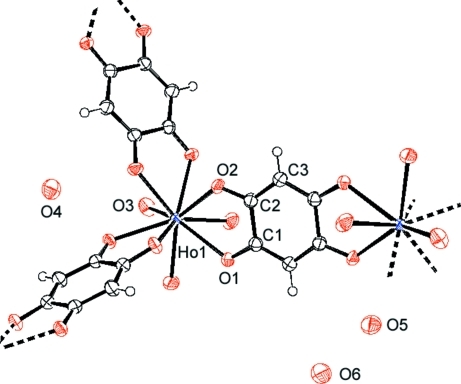
A part of the title polymeric compound, thermal ellipsoids are shown at the 50% probability level. Blue, gray, and red represent Ho, C and O atoms, respectively. The labeled atoms indicate all independent atoms.

**Fig. 2.**
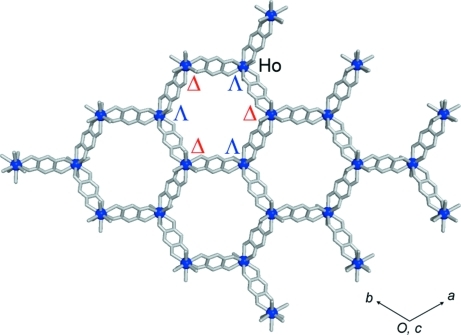
A layer with a honeycomb structure. Blue spheres and gray sticks represent Ho atoms and other atoms (C and O), respectively.

## supplementary crystallographic information

### Comment

Lanthanide complexes have attracted attention as magnetic and luminescent materials due to the properties of the 4*f* orbitals in lanthanide ions. Although the magnetism of mononuclear lanthanide complexes are well understood, studies on polynuclear lanthanide complexes are much less advanced (Ohkoshi *et al.*, 2001). The dimensionality of complexes and coordination geometry around lanthanide ions are key factors to control their magnetic properties. From this viewpoint, constructing polynuclear lanthanide complexes with various topologies are interesting. In our previous work, we reported the 3-D monometallic lanthanide metal assembly, Na_5_[{Ho(THB^4-^)_2_}.7H_2_O]_n_ (THB = 1,2,4,5-tetrahydroxybenzene) (Nakabayashi *et al.*, 2009). In this work, we synthesized a 2-D honeycomb network composed of holmium ions (Ho^III^) and 2,5-dihydroxy-1,4-benzoquinonate, [{Ho_2_ (DHBQ^2-^)_3_ (H_2_O)_6_}.18H_2_O]_n_ (Kitagawa *et al.*, 2002; Robl *et al.*, 1988).

The asymmetric unit comprises a third of a Ho^III^ ion, being located on a three-fold axis, one-half of a DHBQ^2-^ ion, being disposed about a centre of inversion, one coordinated water molecule, and three zeolitic water molecules.

The C—O distances of 1.276 (4) Å (C1—O1) and 1.274 (3) Å (C2—O2) in this compound agree with those of the DHBQ^2-^ ligands which were previously reported, *e.g.* 1.276 (6) Å (Weiss *et al.* 1986). In the coordination geometry, a Ho^III^ ion is coordinated to six O atoms from three bidentate DHBQ^2-^ ligands and three O atoms from three water molecules (Fig. 1 and Table 1). The Ho^III^ ion is connected *via* three ligands, which results in a two-dimensional honeycomb layer with a diameter of 16.6 Å. The layer has a racemic structure in which Δ- and Λ-coordination geometries around Ho^III^ ions are alternately arranged (Fig. 2). The zeolitic water molecules occupy regions between the layers.

The product of the molar magnetic susceptibility (*χ*_M_) and temperature (*T*), *χ*_M_*T*, values at room temperature was 13.6 cm^3^ K mol^-1^. This value nearly corresponds to the expected value of 13.9 cm^3^ K mol^-1^ due to Ho^III^ ions (*J* = 8, *L* = 6, *S* = 2, and *g* = 5/4).

All known compounds of this type have two specific structures, honeycomb or racemic, and show paramagnetism. Mixing lanthanide ions, the chiral lanthanide assemblies with Δ- or Λ-coordination geometries are targeted for synthesis, which should show a magneto-chiral dichroism. A study to clarify this hypotheses, work is currently under way.

### Experimental

Under air, aqueous solutions of 0.1 *M* Ho(NO_3_)_3_ and 0.4 *M* 1,2,4,5-tetrahydroxybenzene (THB) (Weider *et al.*, 1985) mixed. THB was gradually oxidized to 2,5-dihydroxy-1,4-benzoquinone (DHBQ) in the mixed solution, and slow complexation of Ho(NO_3_)_3_ and DHBQ) produced red crystals of the title polymer in 24% yield within a week. The obtained polycrystalline compound was dried under air. Elemental analysis indicated the formula was [Ho_2_(C_6_H_2_O_4_)_3_(H_2_O)_6_].17H_2_O, C_18_H_52_O_35_Ho_2_, calcd. Ho, 28.18%; C, 18.47%; H, 4.49%, found. Ho, 28.29%; C, 18.23%; H, 4.64%. There is a slight difference of zeolitic water molecules between the elemental analysis and the crystallographic formulation because zeolitic water molecules are easy to be lost from the crystals and their number depends on the drying processes.

### Refinement

The H atoms were placed in their calculated positions,with C—H = 0.95 Å, and refined using a riding model, with *U*_iso_(H) = 1.2 *U*_eq_(C).

### Crystal data

**Table:**

[Ho_2_(C_6_H_2_O_4_)_3_(H_2_O)_6_]·18H_2_O	*D*_x_ = 1.796 Mg m^−^^3^
*M**_r_* = 1176.46	Mo *K*α radiation, λ = 0.71075 Å
Trigonal, *R*3	Cell parameters from 9640 reflections
Hall symbol: -R 3	θ = 3.4–27.5°
*a* = 14.1407 (3) Å	µ = 3.88 mm^−^^1^
*c* = 18.0629 (5) Å	*T* = 90 K
*V* = 3127.93 (12) Å^3^	Platelet, red
*Z* = 3	0.10 × 0.10 × 0.04 mm
*F*(000) = 1608.00	

### Data collection

**Table:**

Rigaku R-AXIS RAPID diffractometer	1525 reflections with *F*^2^ > 2σ(*F*^2^)
Detector resolution: 10.00 pixels mm^-1^	*R*_int_ = 0.025
ω scans	θ_max_ = 27.5°
Absorption correction: multi-scan (*ABSCOR*; Higashi, 1995)	*h* = −18→18
*T*_min_ = 0.704, *T*_max_ = 0.856	*k* = −18→18
11262 measured reflections	*l* = −23→23
1594 independent reflections	

### Refinement

**Table:**

Refinement on *F*^2^	H-atom parameters constrained
*R*[*F*^2^ > 2σ(*F*^2^)] = 0.023	*w* = 1/[σ^2^(*F*_o_^2^) + (0.0248*P*)^2^ + 19.995*P*] where *P* = (*F*_o_^2^ + 2*F*_c_^2^)/3
*wR*(*F*^2^) = 0.063	(Δ/σ)_max_ = 0.002
*S* = 1.23	Δρ_max_ = 0.65 e Å^−^^3^
1594 reflections	Δρ_min_ = −0.30 e Å^−^^3^
86 parameters	

### Special details

**Table:**

Geometry. loop_ _Bond lengths and angles Ho1 - Distance Angles O1_$1 2.3715 (0.0023) O1 2.3715 (0.0024) 78.04 (0.09) O1_$2 2.3715 (0.0023) 78.05 (0.09) 78.05 (0.09) O3_$2 2.3851 (0.0025) 138.54 (0.09) 85.02 (0.10) 134.88 (0.09) O3_$1 2.3851 (0.0025) 134.88 (0.09) 138.54 (0.09) 85.02 (0.10) 80.68 (0.11) O3 2.3852 (0.0025) 85.02 (0.10) 134.88 (0.09) 138.54 (0.09) 80.68 (0.11) 80.68 (0.11) O2_$1 2.4626 (0.0024) 65.01 (0.08) 134.82 (0.08) 69.90 (0.08) 140.07 (0.09) 69.92 (0.09) 68.65 (0.09) O2 2.4626 (0.0023) 69.90 (0.08) 65.01 (0.08) 134.82 (0.08) 68.65 (0.09) 140.07 (0.09) 69.92 (0.08) 119.91 (0.01) O2_$2 2.4626 (0.0023) 134.82 (0.08) 69.90 (0.08) 65.01 (0.08) 69.92 (0.08) 68.65 (0.09) 140.07 (0.09) 119.91 (0.01) Ho1 - O1_$1 O1 O1_$2 O3_$2 O3_$1 O3 O2_$1 O1 - Distance Angles C1 1.2764 (0.0041) Ho1 2.3715 (0.0023) 123.38 (0.21) O1 - C1 O2 - Distance Angles C2 1.2739 (0.0041) Ho1 2.4626 (0.0023) 119.92 (0.21) O2 - C2 O3 - Distance Angles Ho1 2.3852 (0.0025) O3 - C1 - Distance Angles O1 1.2764 (0.0041) C3 1.3846 (0.0049) 125.21 (0.32) C2 1.5290 (0.0046) 114.26 (0.29) 120.50 (0.30) C1 - O1 C3 C3 - Distance Angles C1 1.3846 (0.0049) C2_$3 1.3982 (0.0048) 119.67 (0.32) H3 0.9500 120.16 120.16 C3 - C1 C2_$3 C2 - Distance Angles O2 1.2739 (0.0041) C3_$3 1.3982 (0.0048) 124.89 (0.31) C1 1.5290 (0.0046) 115.30 (0.29) 119.80 (0.30) C2 - O2 C3_$3
Refinement. Refinement was performed using all reflections. The weighted *R*-factor (*wR*) and goodness of fit (*S*) are based on *F*^2^. *R*-factor (gt) are based on *F*. The threshold expression of *F*^2^ > 2.0 σ(*F*^2^) is used only for calculating *R*-factor (gt).

### Fractional atomic coordinates and isotropic or equivalent isotropic displacement parameters (Å^2^)

**Table:**

	*x*	*y*	*z*	*U*_iso_*/*U*_eq_	
Ho1	0.0000	0.0000	0.754297 (13)	0.01802 (8)	
O1	−0.1198 (2)	−0.12391 (19)	0.66415 (13)	0.0270 (5)	
O2	−0.00678 (19)	−0.17736 (19)	0.75023 (13)	0.0252 (4)	
O3	0.1248 (2)	−0.0026 (2)	0.84201 (15)	0.0366 (6)	
O4	0.1390 (2)	−0.1541 (2)	0.93023 (18)	0.0481 (7)	
O5	0.2672 (2)	0.1817 (2)	0.92192 (19)	0.0508 (7)	
O6	−0.1882 (2)	−0.0598 (2)	0.54192 (17)	0.0474 (7)	
C1	−0.1492 (2)	−0.2252 (2)	0.66312 (19)	0.0246 (6)	
C3	−0.2332 (2)	−0.3039 (2)	0.6206 (2)	0.0283 (7)	
C2	−0.0802 (2)	−0.2555 (2)	0.71218 (18)	0.0237 (6)	
H3	−0.2770	−0.2850	0.5912	0.034*	

### Atomic displacement parameters (Å^2^)

**Table:**

	*U*^11^	*U*^22^	*U*^33^	*U*^12^	*U*^13^	*U*^23^
Ho1	0.01630 (11)	0.01630 (11)	0.02146 (14)	0.00815 (5)	0.0000	0.0000
O1	0.0310 (13)	0.0178 (11)	0.0320 (12)	0.0121 (10)	−0.0067 (9)	−0.0020 (9)
O2	0.0240 (11)	0.0194 (11)	0.0316 (12)	0.0103 (9)	−0.0047 (9)	−0.0032 (9)
O3	0.0363 (14)	0.0309 (14)	0.0405 (14)	0.0154 (12)	−0.0164 (11)	−0.0020 (11)
O4	0.0455 (18)	0.0513 (18)	0.0552 (18)	0.0299 (15)	−0.0062 (14)	0.0051 (15)
O5	0.0490 (18)	0.0453 (18)	0.0558 (19)	0.0218 (15)	−0.0121 (15)	−0.0078 (14)
O6	0.0528 (19)	0.0470 (18)	0.0418 (16)	0.0244 (15)	−0.0105 (13)	0.0015 (13)
C1	0.0241 (16)	0.0227 (15)	0.0271 (15)	0.0116 (13)	0.0016 (12)	0.0014 (12)
C3	0.0274 (17)	0.0235 (17)	0.0334 (17)	0.0122 (14)	−0.0049 (13)	0.0001 (13)
C2	0.0228 (15)	0.0236 (16)	0.0241 (15)	0.0111 (13)	0.0019 (12)	0.0005 (12)

### Geometric parameters (Å, °)

**Table:**

Ho1—O1	2.371 (2)	Ho1—O3^ii^	2.385 (2)
Ho1—O1^i^	2.371 (2)	O1—C1	1.276 (4)
Ho1—O1^ii^	2.371 (2)	O2—C2	1.274 (3)
Ho1—O2	2.463 (2)	C1—C3	1.385 (4)
Ho1—O2^i^	2.4625 (19)	C1—C2	1.529 (6)
Ho1—O2^ii^	2.463 (3)	C3—C2^iii^	1.398 (5)
Ho1—O3	2.385 (3)	C3—H3	0.950
Ho1—O3^i^	2.385 (3)		
			
Ho1···C1	3.253 (3)	O6···C1	3.444 (5)
Ho1···C1^i^	3.253 (3)	O6···C1^ii^	3.369 (4)
Ho1···C1^ii^	3.253 (4)	O6···C3	3.485 (5)
Ho1···C2	3.289 (3)	O6···C3^xi^	3.326 (5)
Ho1···C2^i^	3.289 (2)	C1···Ho1	3.253 (3)
Ho1···C2^ii^	3.289 (4)	C1···O1^i^	3.592 (3)
O1···O1^i^	2.986 (3)	C1···O2	2.372 (4)
O1···O1^ii^	2.986 (3)	C1···O3^ii^	3.481 (4)
O1···O2	2.599 (4)	C1···O6	3.444 (5)
O1···O2^ii^	2.770 (4)	C1···O6^i^	3.369 (4)
O1···O3^ii^	3.214 (3)	C1···C1^iii^	2.847 (5)
O1···O4^iv^	3.464 (4)	C1···C3^iii^	2.533 (6)
O1···O6	2.740 (4)	C1···C2^iii^	2.406 (4)
O1···O6^i^	3.390 (4)	C3···O1	2.363 (3)
O1···C1^ii^	3.592 (5)	C3···O2^iii^	2.370 (4)
O1···C3	2.363 (3)	C3···O6	3.485 (5)
O1···C2	2.360 (5)	C3···O6^xii^	3.326 (4)
O1···C2^ii^	3.458 (5)	C3···C1^iii^	2.533 (6)
O2···O1	2.599 (4)	C3···C3^iii^	2.927 (6)
O2···O1^i^	2.770 (3)	C3···C2	2.531 (5)
O2···O3	2.778 (3)	C2···Ho1	3.289 (3)
O2···O3^ii^	2.734 (4)	C2···O1	2.360 (5)
O2···O4^v^	2.868 (5)	C2···O1^i^	3.458 (3)
O2···O5^ii^	3.325 (4)	C2···O3^ii^	3.254 (5)
O2···C1	2.372 (4)	C2···O4^v^	3.499 (5)
O2···C3^iii^	2.370 (4)	C2···C1^iii^	2.406 (4)
O3···O1^i^	3.214 (3)	C2···C3	2.531 (5)
O3···O2	2.778 (3)	C2···C2^iii^	2.855 (4)
O3···O2^i^	2.734 (3)	O1···H3	2.608
O3···O3^i^	3.088 (5)	O2···H3^iii^	2.614
O3···O3^ii^	3.088 (4)	O4···H3^ix^	3.259
O3···O4	2.757 (5)	O4···H3^vi^	3.484
O3···O5	2.772 (3)	O5···H3^ix^	3.135
O3···C1^i^	3.481 (4)	O6···H3	2.918
O3···C2^i^	3.254 (3)	O6···H3^xi^	3.032
O4···O1^vi^	3.464 (5)	C1···H3	2.035
O4···O2^v^	2.868 (5)	C1···H3^iii^	3.400
O4···O3	2.757 (5)	C2···H3	3.396
O4···O5^ii^	2.753 (4)	C2···H3^iii^	2.048
O4···O5^vii^	2.802 (4)	H3···O1	2.608
O4···C2^v^	3.499 (5)	H3···O2^iii^	2.614
O5···O2^i^	3.325 (4)	H3···O4^x^	3.259
O5···O3	2.772 (3)	H3···O4^iv^	3.484
O5···O4^i^	2.753 (6)	H3···O5^x^	3.135
O5···O4^viii^	2.802 (4)	H3···O6	2.918
O5···O6^ix^	2.728 (4)	H3···O6^xii^	3.032
O6···O1	2.740 (4)	H3···C1	2.035
O6···O1^ii^	3.390 (3)	H3···C1^iii^	3.400
O6···O5^x^	2.728 (4)	H3···C2	3.396
O6···O6^xi^	2.801 (4)	H3···C2^iii^	2.048
O6···O6^xii^	2.801 (5)		
			
O1—Ho1—O1^i^	78.04 (8)	O2—Ho1—O3^i^	140.07 (9)
O1—Ho1—O1^ii^	78.04 (8)	O2—Ho1—O3^ii^	68.65 (10)
O1—Ho1—O2	65.01 (9)	O2^i^—Ho1—O2^ii^	119.91 (9)
O1—Ho1—O2^i^	134.81 (7)	O2^i^—Ho1—O3	68.65 (8)
O1—Ho1—O2^ii^	69.90 (9)	O2^i^—Ho1—O3^i^	69.92 (8)
O1—Ho1—O3	134.88 (10)	O2^i^—Ho1—O3^ii^	140.07 (8)
O1—Ho1—O3^i^	138.54 (11)	O2^ii^—Ho1—O3	140.07 (9)
O1—Ho1—O3^ii^	85.02 (8)	O2^ii^—Ho1—O3^i^	68.65 (9)
O1^i^—Ho1—O1^ii^	78.04 (10)	O2^ii^—Ho1—O3^ii^	69.92 (10)
O1^i^—Ho1—O2	69.90 (8)	O3—Ho1—O3^i^	80.68 (11)
O1^i^—Ho1—O2^i^	65.01 (8)	O3—Ho1—O3^ii^	80.68 (9)
O1^i^—Ho1—O2^ii^	134.81 (8)	O3^i^—Ho1—O3^ii^	80.68 (9)
O1^i^—Ho1—O3	85.02 (10)	Ho1—O1—C1	123.4 (2)
O1^i^—Ho1—O3^i^	134.88 (7)	Ho1—O2—C2	119.9 (2)
O1^i^—Ho1—O3^ii^	138.54 (10)	O1—C1—C3	125.2 (4)
O1^ii^—Ho1—O2	134.81 (8)	O1—C1—C2	114.3 (2)
O1^ii^—Ho1—O2^i^	69.90 (8)	C3—C1—C2	120.5 (3)
O1^ii^—Ho1—O2^ii^	65.01 (8)	C1—C3—C2^iii^	119.7 (4)
O1^ii^—Ho1—O3	138.54 (7)	O2—C2—C1	115.3 (3)
O1^ii^—Ho1—O3^i^	85.02 (9)	O2—C2—C3^iii^	124.9 (3)
O1^ii^—Ho1—O3^ii^	134.88 (11)	C1—C2—C3^iii^	119.8 (2)
O2—Ho1—O2^i^	119.91 (9)	C1—C3—H3	120.2
O2—Ho1—O2^ii^	119.91 (7)	C2^iii^—C3—H3	120.2
O2—Ho1—O3	69.92 (9)		
			
O1—Ho1—O1^i^—C1^i^	166.8 (3)	O3—Ho1—O1^ii^—C1^ii^	−125.1 (2)
O1^i^—Ho1—O1—C1	86.7 (2)	O3^i^—Ho1—O1^ii^—C1^ii^	−55.3 (2)
O1—Ho1—O1^ii^—C1^ii^	86.7 (2)	O3^ii^—Ho1—O1^ii^—C1^ii^	16.3 (2)
O1^ii^—Ho1—O1—C1	166.8 (2)	O2—Ho1—O2^i^—C2^i^	34.3 (2)
O1—Ho1—O2—C2	−10.8 (2)	O2^i^—Ho1—O2—C2	−139.8 (2)
O2—Ho1—O1—C1	13.5 (2)	O2—Ho1—O2^ii^—C2^ii^	−139.8 (2)
O1—Ho1—O2^i^—C2^i^	−49.0 (3)	O2^ii^—Ho1—O2—C2	34.3 (2)
O2^i^—Ho1—O1—C1	121.7 (2)	O3—Ho1—O2—C2	171.3 (2)
O1—Ho1—O2^ii^—C2^ii^	−96.6 (2)	O3^i^—Ho1—O2—C2	126.2 (2)
O2^ii^—Ho1—O1—C1	−125.7 (2)	O3^ii^—Ho1—O2—C2	83.8 (2)
O3—Ho1—O1—C1	16.3 (3)	O2^i^—Ho1—O2^ii^—C2^ii^	34.3 (2)
O3^i^—Ho1—O1—C1	−125.1 (2)	O2^ii^—Ho1—O2^i^—C2^i^	−139.8 (2)
O3^ii^—Ho1—O1—C1	−55.3 (2)	O3—Ho1—O2^i^—C2^i^	83.8 (2)
O1^i^—Ho1—O1^ii^—C1^ii^	166.8 (2)	O3^i^—Ho1—O2^i^—C2^i^	171.3 (2)
O1^ii^—Ho1—O1^i^—C1^i^	86.7 (3)	O3^ii^—Ho1—O2^i^—C2^i^	126.2 (2)
O1^i^—Ho1—O2—C2	−96.6 (2)	O3—Ho1—O2^ii^—C2^ii^	126.2 (2)
O2—Ho1—O1^i^—C1^i^	−125.7 (3)	O3^i^—Ho1—O2^ii^—C2^ii^	83.8 (2)
O1^i^—Ho1—O2^i^—C2^i^	−10.8 (2)	O3^ii^—Ho1—O2^ii^—C2^ii^	171.3 (2)
O2^i^—Ho1—O1^i^—C1^i^	13.5 (3)	Ho1—O1—C1—C3	167.6 (2)
O1^i^—Ho1—O2^ii^—C2^ii^	−49.0 (2)	Ho1—O1—C1—C2	−14.4 (4)
O2^ii^—Ho1—O1^i^—C1^i^	121.7 (3)	Ho1—O2—C2—C1	8.1 (3)
O3—Ho1—O1^i^—C1^i^	−55.3 (3)	Ho1—O2—C2—C3^iii^	−171.9 (2)
O3^i^—Ho1—O1^i^—C1^i^	16.3 (3)	O1—C1—C3—C2^iii^	176.2 (3)
O3^ii^—Ho1—O1^i^—C1^i^	−125.1 (3)	O1—C1—C2—O2	3.4 (4)
O1^ii^—Ho1—O2—C2	−49.0 (2)	O1—C1—C2—C3^iii^	−176.5 (3)
O2—Ho1—O1^ii^—C1^ii^	121.7 (2)	C3—C1—C2—O2	−178.4 (3)
O1^ii^—Ho1—O2^i^—C2^i^	−96.6 (2)	C3—C1—C2—C3^iii^	1.7 (5)
O2^i^—Ho1—O1^ii^—C1^ii^	−125.7 (2)	C2—C1—C3—C2^iii^	−1.7 (5)
O1^ii^—Ho1—O2^ii^—C2^ii^	−10.8 (2)	C1—C3—C2^iii^—O2^iii^	−178.5 (3)
O2^ii^—Ho1—O1^ii^—C1^ii^	13.5 (2)	C1—C3—C2^iii^—C1^iii^	1.6 (5)

Symmetry codes: (i) −*y*, *x*−*y*, *z*; (ii) −*x*+*y*, −*x*, *z*; (iii) −*x*−1/3, −*y*−2/3, −*z*+4/3; (iv) *x*−*y*−2/3, *x*−1/3, −*z*+5/3; (v) −*x*+1/3, −*y*−1/3, −*z*+5/3; (vi) *y*+1/3, −*x*+*y*−1/3, −*z*+5/3; (vii) *y*, −*x*+*y*, −*z*+2; (viii) *x*−*y*, *x*, −*z*+2; (ix) *x*+2/3, *y*+1/3, *z*+1/3; (x) *x*−2/3, *y*−1/3, *z*−1/3; (xi) *y*, −*x*+*y*, −*z*+1; (xii) *x*−*y*, *x*, −*z*+1.
